# Intrathecal complement activation by the classical pathway in tick-borne encephalitis

**DOI:** 10.1007/s13365-019-00734-1

**Published:** 2019-03-08

**Authors:** Malin Veje, Marie Studahl, Tomas Bergström

**Affiliations:** Department of Infectious Diseases, Institute of Biomedicine, Sahlgrenska Academy, University of Gothenburg, Gothenburg, Sweden

**Keywords:** Tick-borne encephalitis (TBE), Complement, C1q, Intrathecal activation

## Abstract

Tick-borne encephalitis (TBE) is one of the most prevalent viral central nervous system (CNS) infections in Eurasia and neurological sequelae are common. The immune responses are considered crucial for the pathogenesis. The aim of this study was to explore the activation of the complement system in TBE. The complement system is a part of the innate immune response in the CNS, which previously has been reported to be activated in other flavivirus infections. We analyzed complement factors in 44 paired cerebrospinal fluid (CSF) and serum samples from 20 cases of TBE in the acute and later stages, as well as in serum and CSF from 32 healthy controls. The concentrations of complement factors C1q, C3a, C3b, and C5a were determined with commercially available ELISA kits. Clinical data to categorize the severity of disease and outcome was retrieved from the medical records of the TBE patients. We found significantly higher concentrations of all of the analyzed complement factors in the CSF from TBE patients compared to the healthy controls. In particular, the marked increment of C1q concentrations in the CSF (*p* < 0,001 as compared to controls) indicated an intrathecal activation by the classical pathway. There was no correlation between complement factor concentrations in the CSF and severity of the disease in the acute phase or with sequelae at 6 months follow-up. We have found an intrathecal complement activation in TBE, and the marked increase of complement factor C1q indicated an activation by the classical pathway.

## Introduction

Tick-borne encephalitis (TBE) is one of the most prevalent viral central nervous system (CNS) infections in Eurasia and neurological sequelae are common in adults as well as in children. The TBE virus (TBEV) belongs to the genus *Flavivirus*, other important human pathogenic viruses in this group being dengue, yellow fever, Zika, Japanese encephalitis, and West Nile viruses. TBEV is spread by ticks, with rodents as intermediate hosts (Lindquist and Vapalahti 2008). Findings of TBEV RNA in cerebrospinal fluid (CSF) are rare (Saksida et al. 2005; Veje et al. 2018), but viral RNA has been detected in the brain in fatal cases (Kuivanen et al. 2015; Tomazic et al. 1997). Although the host immune response to the virus is considered to be a major contributor to the CNS tissue destruction in TBE, such reactions have not been fully characterized (Růžek et al. 2009).

**Fig. 1:**
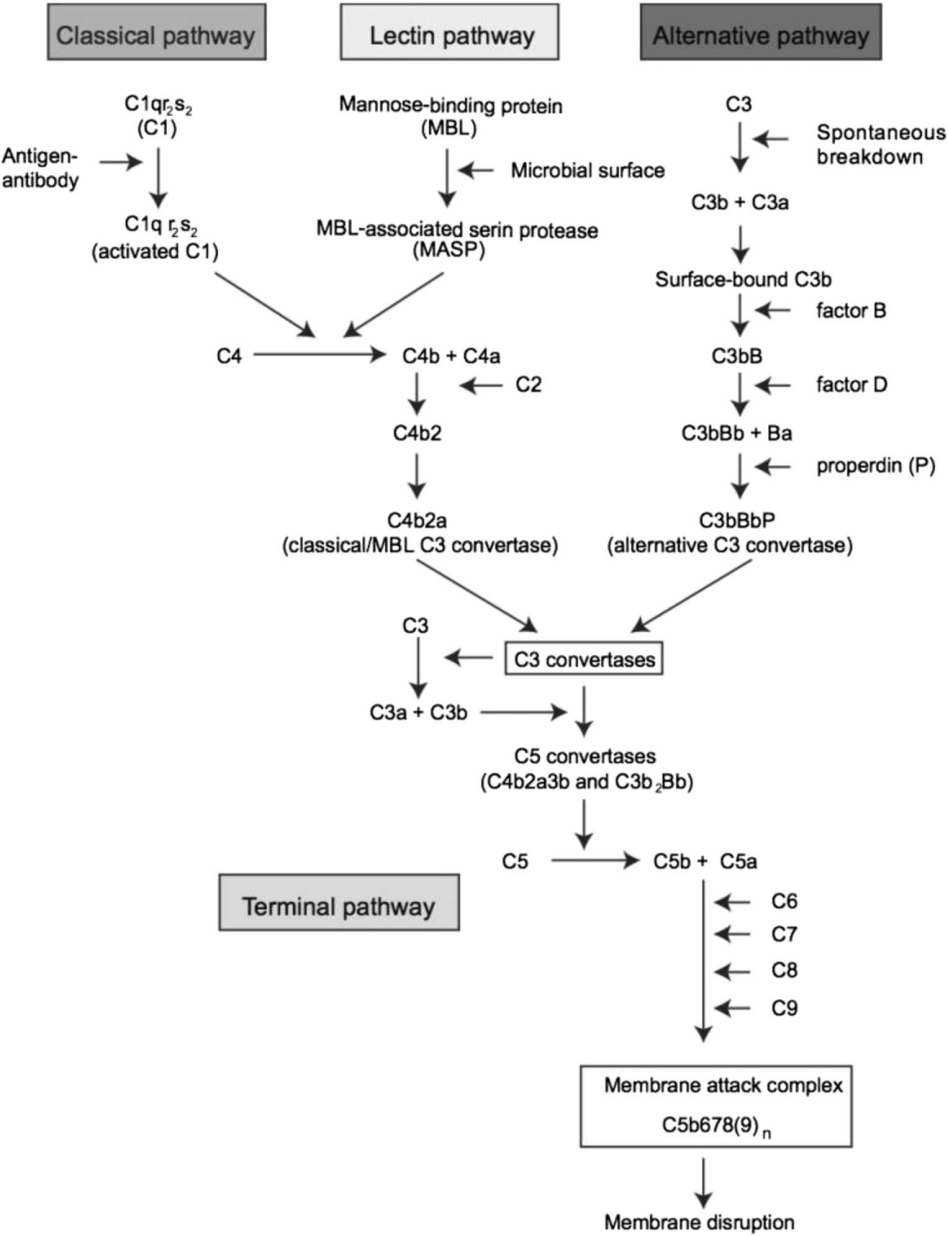
Overview of the three major complement system activation pathways and the terminal pathway. Reproduced with permission from Charlotta Eriksson (Thesis, Gothenburg University, 2016)

The complement system constitutes a part of the innate immune system, and is mainly activated by three routes: the Classical, the Lectin, and the Alternative pathways (Fig. 1). As a reaction to pathogens and trauma, several different proteins are produced and take part in a cascade of activation steps, leading to a direct immunological response as well as activation of the adaptive immune system (Shastri et al. 2013). Complement activation in the CNS has been most extensively studied in the host defense against bacterial and fungal infections (Shen et al. 2017; Mook-Kanamori et al. 2014; Henningsson et al. 2007). In viral infections, we have reported that C3a, C3b, C5, and C5a increased intrathecally in the acute stage in patients with herpes simplex encephalitis (HSE) and that C3a and C5a remained increased in later stages, suggesting that complement activation may also be a part of a chronic neuroinflammation (Eriksson et al. 2016). There has been an interest in the role of complement activation in another flavivirus infection of the CNS, West Nile virus (Mehlhop et al. 2007; Mehlhop and Diamond 2006; Mehlhop et al. 2009a; Mehlhop et al. 2009b; Mehlhop et al. 2005). In cell cultures and in mice models, complement activation is required for antibody-mediated neutralization, and especially C1q seems to have a crucial role for this function (Mehlhop et al. 2007; Mehlhop et al. 2009b).

The role of complement activation for the pathogenesis of TBE has not been established. To this end, the aim of our study was to determine concentrations of the complement activation factors C1q, C3a, C3b, and C5a, in serum and CSF in samples from TBE patients and compare those to samples from healthy controls. Here, we found a strong intrathecal complement activation as part of the innate immune response against TBEV.

## Materials and methods

### TBE patients

Serum and cerebrospinal fluid (CSF) samples were drawn from prospectively included patients with TBE at the Department of Infectious Diseases, Sahlgrenska University Hospital, during the period 2014–2017. All TBE patients above 18 years of age, admitted to the inpatient clinic or referred to the outpatient clinic, were asked to join the study. There were no exclusion criteria except inability to give oral and written consent (patient and/or next of kin). All cases had positive TBEV–specific IgM and IgG antibodies in serum (Enzygnost Anti-TBE Virus (IgG, IgM), Siemens) and were confirmed according to the ECDC definition (ECDC Meeting Report 2011 2012) from 2011, where a confirmed TBE case requires both clinical symptoms of TBE *and* IgM plus IgG in serum *or* IgM in the CSF *or* IgM plus IgG in the CSF *or* detection of TBE viral nucleic acid in clinical specimen. Patient samples with both serum and CSF from the same date were included in the study.

The medical records were studied and a clinical classification of the severity of the symptoms in the acute phase, i.e. “mild,” “moderate,” or “severe,” was performed, according to our previous study (Veje et al. 2016). Mild disease was defined as primarily meningeal symptoms like fever, headache, nausea, vomiting, neck stiffness, and sensitivity to light and sound with a normal EEG or EEG not performed. Moderate disease was defined as moderate signs of encephalitis without or with slightly altered consciousness, and/or diffuse neurological symptoms such as confusion, slow thinking, or focal neurological symptoms such as ataxia, tremor, or dysphasia. Severe disease was defined as multifocal symptoms and/or severe signs of encephalitis with altered consciousness.

The medical records at the clinical follow-ups were analyzed, and the patients were divided into two groups, “fully recovered” and “sequelae,” based on data from clinical examinations after approximately 3, 6, and 12 months.

### Healthy controls

Frozen paired CSF and serum biobank samples, drawn from 32 healthy control persons in a previous study (Persson et al. 2014), were thawed at room temperature and analyzed for complement factors. These subjects were recruited by advertisement at the Center of Blood Donation at the Sahlgrenska University Hospital.

### Determination of complement factors

The concentrations of the complement factors C1q, C3a, C3b, and C5a were analyzed in serum and CSF with commercially available ELISA kits, according to the instructions supplied by the manufacturers (Cusabio Biotech for C3b and Cloud-Clone Corp. for the others). The respective kit sensitivity and specificity was provided by the producers, as previously described (Eriksson et al. 2016).

### Statistical analysis

GraphPad Prism version 7.0 (GraphPad Software, La Jolla California, USA) was used for statistical analyses and graph constructions. Comparisons were made by the Mann-Whitney *U* test, where a *p* value < 0.05 was considered significant.

## Results

### Patients and controls

The control group was younger than the TBE patient group and consisted of a higher proportion of men. There was a wider age range in the TBE group (34–88 years) compared to the control group (18–57 years) (Table 1). According to the definition above, four patients (20%) had a mild TBE disease, 15 patients (75%) a moderate, and one patient (5%) suffered from a severe disease. For each patient, one (*n* = 5), two (*n* = 6) or three (*n* = 9) paired CSF/serum samples were available. All in all, 44 paired samples were analyzed for complement factors, drawn with a range from 0 to 327 days after fever onset, four samples on days 0–7, 24 samples on days 8–31, nine samples on days 32–90, and seven samples were drawn on days 91–327 after fever onset. 42/44 of the paired serum CSF samples were drawn on the same day, while 2/44 within 1 day apart. During the study period, 19 TBE patients were admitted to the inpatient clinic. Of these, 16 were included in the study and three of the study patients were included after referral from other departments (e.g., internal medicine, neurology) to our outpatient clinic. One of the patients was hospitalized with TBE in 2012 but was not referred to the outpatient clinic until 2016, when he was included in the study.

**Table 1:** Characteristics of the included tick-borne encephalitis (TBE) patients and controls

	TBE patients (*n* = 20)	Control persons (*n* = 32)
Median age, years (range)	52.5 (34–88)	30 (18–57)
Male gender	11 (55%)	20 (62.5%)
Concomitant diseases*	9/20 (45%)	–
Number of CSF samples	44	32
Number of serum samples	44	32

^*^11/20 patients were previously completely healthy. Of the 9 patients with concomitant diseases, 2/9 had substitution treatment for hypothyroid, 2/9 were treated for hypertonia, 2/9 had treatment for depression and/or bipolar disease, 6/9 had musculoskeletal pain, 1/9 was operated for mammalian cancer, 1/9 was operated for colon cancer, 1/9 was treated with estrogen substitution, and 1/9 had chronic obstructive lung disease. Neither of the patients were on immunosuppressive treatment

### Complement activation in TBE patients

**Fig. 2:**
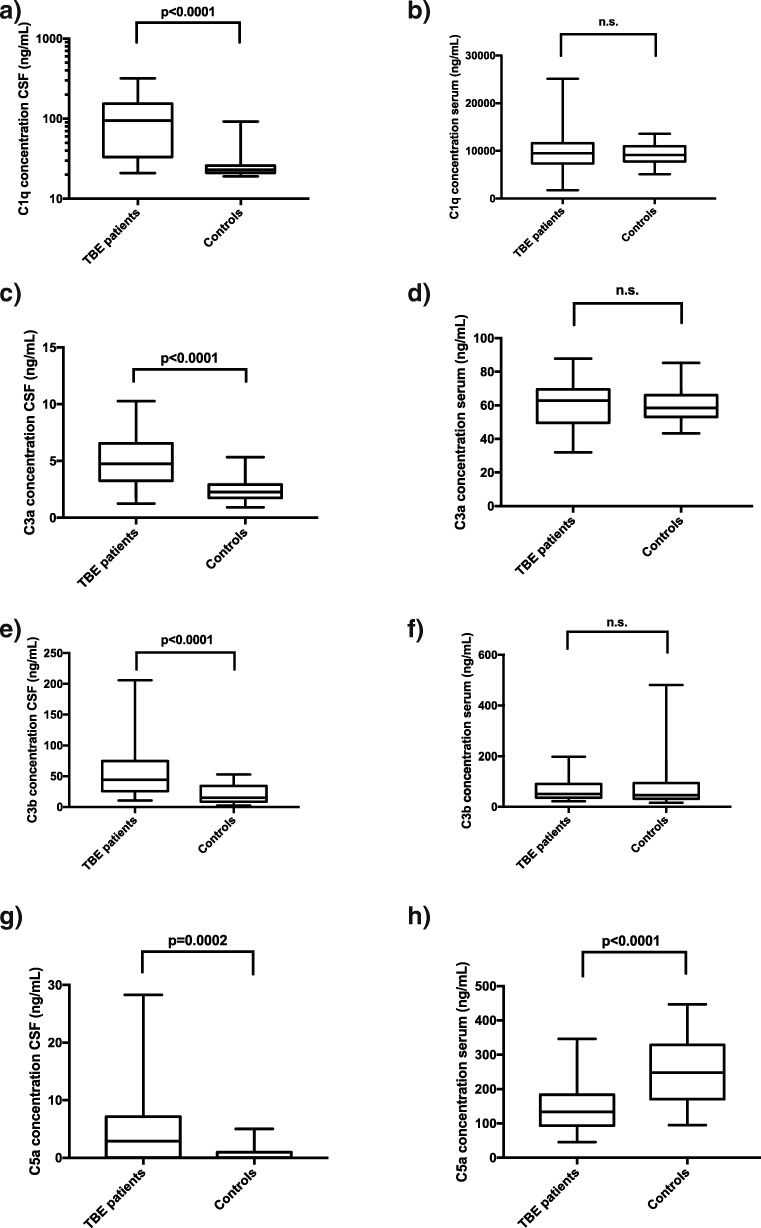
Box and whiskers plot displaying a comparison of complement factor C1q, C3a, C3b, and C5a concentrations (ng/mL) in cerebrospinal fluid (CSF) and serum samples, measured with enzyme-linked immunosorbent assays (ELISAs), from TBE patients and controls. Lines showing medians and interquartile ranges, whiskers at minimum and maximum values

Compared to the controls, the TBE patients had significantly higher levels of all of the analyzed complement factors in the CSF (Fig. 2). C1q, a complement factor part of the classical pathway, was detected in higher concentrations than C3a, C3b, and C5a. Regarding the serum samples, the complement factor concentrations were generally higher in the patient than in the control group, but there was no statistical difference between the patient and the control group, except for the C5a concentrations, which were statistically higher in the controls than in the sera from the TBE group.

### Complement activation in relation to disease severity and age

Since the vast majority of the patients (15/20) suffered from a moderate disease, correlation between disease severity and complement factor concentrations could not be properly investigated. One patient had markedly higher C3b concentrations in the CSF compared to the other patients. This was a 65-year-old male, suffering from a paresis in his right arm and hand, without improvement at the 12-months´ -follow-up, despite intense rehabilitation. The C5a concentration in the CSF was markedly higher in one 40-year-old female, compared to the other patients. She was 30 weeks pregnant with twins and suffered from severe encephalitis, with incomplete recovery after 12 months.

We found no correlation between the patients’ age and complement factor concentrations in the first CSF sample (drawn on days 7–79 (median 19.5) after fever onset) or between complement activation and intrathecal mononuclear pleocytosis in the first CSF sample (data not shown).

### Kinetics of complement activity in acute and later stages of TBE

**Fig. 3:**
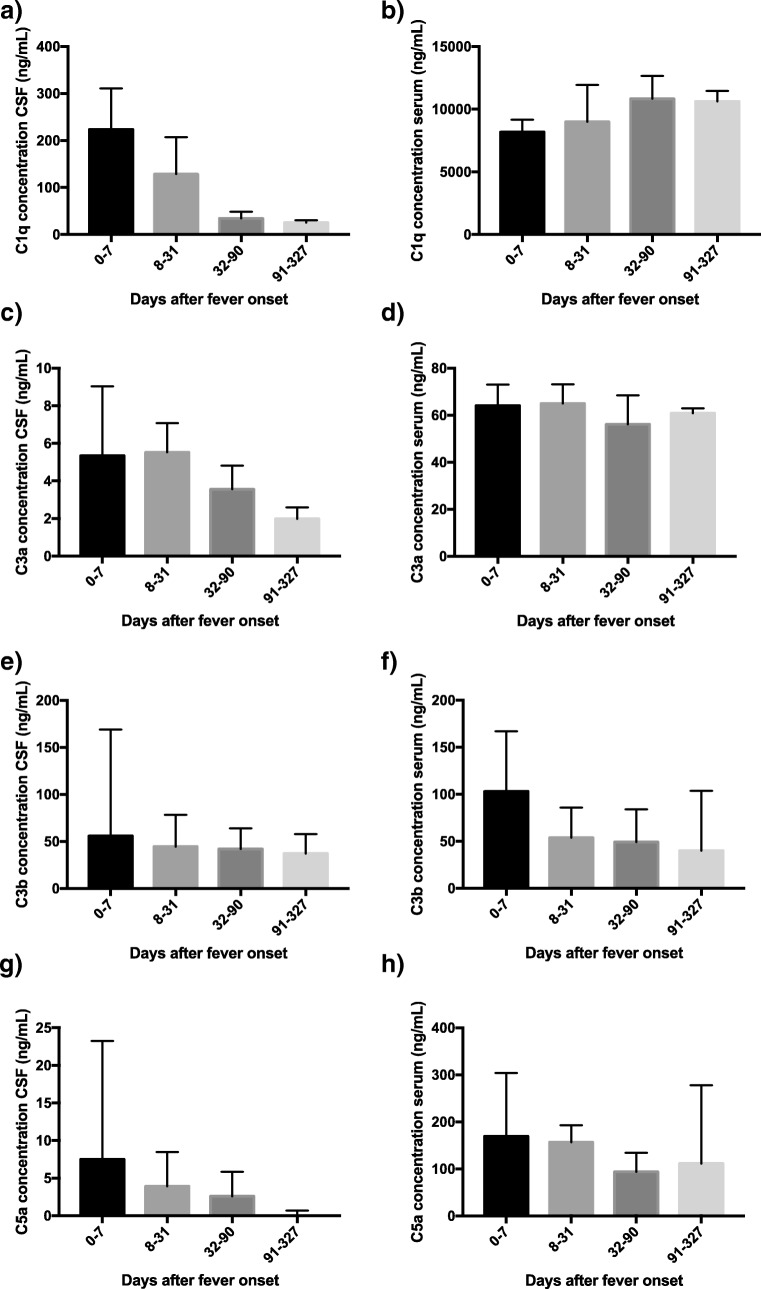
Box and whiskers plot of distributions of cerebrospinal fluid (CSF) and serum concentrations (ng/mL) of complement factors C1q, C3a, C3b, and C5a, measured with enzyme-linked immunosorbent assays (ELISAs), in TBE patients at different time points after the infection. Days 0–7: number of samples, *n* = 4, days 8–31: *n* = 24, days 32–90: *n* = 9, days 91–327: *n* = 7. Median concentrations are presented for each time period, with interquartile ranges indicated with error bars

The kinetics of the different complement factors, shown for paired samples of CSF and serum for the time periods of 0–7, 8–31, 32–90, and 91–327 days after fever onset, are found in Fig. 3. In serum, the concentrations of complement factors were rather stable over time and the concentrations were generally around tenfold higher in serum than in the CSF. Intrathecally, the complement concentrations diminished rapidly within the first 3 months after the infection, this pattern being most pronounced in the C1q concentrations, which were higher than the concentrations of C3a, C3b, and C5a. More than 3 months after disease onset, there was still detectable intrathecal complement activation of the complement factors C1q and C3b (Fig. 3a, e).

### Complement activation in relation to sequelae

Medical records regarding symptoms at minimum 3 months follow-up (range 3–6 months) were available in 14/20 patients. One patient was lost to follow-up, three patients had withdrawn from the study, one patient was temporarily lost to follow-up and had his first revisit 10 months after the infection, and one patient had no follow-up visit at 6 months because he was included 4 years after the infection and saved samples from the acute phase were analyzed. In 14/20 patients, we had follow-up data in minimum 6 months after disease onset (range 6.5–11 months), and follow-up data 12 months after the infection (range 12–17 months) was available in 10/20 patients. At 3 months, 12/14 (85.7%) of the patients still experienced symptoms, at 6 months 10/14 (71.4%), and 1 year after the onset of disease 9/10 patients (90%) still suffered from sequelae.

We compared the intrathecal complement factor concentrations between the fully recovered patients with the patients suffering from sequelae at the different follow-up time points (3, 6, and 12 months), and found no statistical difference between the groups, except for at the 3 months follow-up, where the fully recovered patients had significantly higher levels of the complement factor C3b compared to the patients with sequelae (data not shown).

## Discussion

Given the limited data available on intrathecal complement activation in viral CNS infections in general, and in TBE in particular, our study may increase understanding of the innate CNS immune response to this virus. Our samples were drawn at different stages of the disease, which gave us the opportunity to study complement activation at various time points during the disease course. It was evident that the intrathecal activation of the complement system was strong and rapid in patients with TBE, and that, based on high levels of complement factor C1q in the CSF, the classical pathway was utilized.

The immune response against TBEV involves several components. Serum levels of cytokines are elevated in TBE patients compared to controls (Palus et al. 2015). In mouse models, increased permeability of the blood-brain-barrier in TBEV infection is preceded by high levels of cytokines and chemokines in the brain (Ruzek et al. 2011). Neopterin and beta 2 microglobulin are produced by monocytes and macrophages in response to infection and are proposed to be surrogate markers for T cell and lymphocyte activity (Gunther et al. 1996).

Although the understanding of intrathecal complement activation is incomplete, previous studies have found that several complement factors were upregulated in CNS infections and other brain diseases (Shen et al. 2017; Michailidou et al. 2015; Ager et al. 2010; Kasanmoentalib et al. 2017; Hellewell 2012). The clinical relevance of complement activation might have several facets. For example, C3a added to the nasal mucosa of mice can decrease the neurological sequelae after ischemic stroke (Stokowska et al. 2017). C1q and the classical complement pathway have been found to be important in Alzheimer’s disease as well as in normal aging (Stephan et al. 2013). C5a is a pro-inflammatoric substance and has been connected to several conditions, such as inflammatory disorders and sepsis (Guo and Ward 2005). Elevated levels of complement factors in plasma have been detected in pregnant women (Richani et al. 2005). The patients in our study were older than the healthy controls, which might have influenced the results. However, our study did not detect any correlation between age and complement factor concentration, presumably due to the small sample size.

Infection with West Nile virus (WNV), a mosquito-borne flavivirus with genetic similarity to TBEV, is known to elicit complement activation. This has been shown both in in vitro and in animal studies (Mehlhop et al. 2007; Mehlhop and Diamond 2006; Mehlhop et al. 2009a; Mehlhop et al. 2009b; Mehlhop et al. 2005). In a mouse model of WNV infection, complement factor C1q appeared to boost the WNV antibody activity, and in the presence of C1q, the number of WNV antibodies needed to neutralize the virus was lowered (Mehlhop et al. 2009b). Dengue virus (DENV), another flavivirus, also activates the complement system (Conde et al. 2017). In the present study, significantly higher C1q concentrations were detected in TBE patients than in healthy controls. Furthermore, the here reported C1q concentrations where higher than both the C3a, C3b, and C5a concentrations. The finding that complement factor C1q may play an important role in the immune response to TBEV is in line with data from previous studies on WNV and DENV (Mehlhop et al. 2007; Mehlhop and Diamond 2006; Mehlhop et al. 2009b; Conde et al. 2017). Interestingly, factor C5a concentrations were lower in peripheral blood in patients compared to controls, possibly explained by binding to leukocyte receptors as a control mechanism against systemic effects (Oppermann and Gotze 1994). The majority of TBE patients have leukocytosis in peripheral blood when neurological symptoms are present (Kaiser 1999) and leukocytosis is a prerequisite for removal of C5a from the blood (Oppermann and Gotze 1994).

No correlation was found between intrathecal complement factor concentrations and sequelae. Due to drop-out during follow-up in the study, we could only retrieve 12 months´ data on 10/20 patients, and only one of those patients was fully recovered. This might suggest a bias of drop-out from the study, where the recovered patients were less motivated to be followed up in the study. Perhaps this potential bias could also influence the difference in complement factor concentrations between patients and controls. However, both the patient who was fully recovered at 12 months, and a preponderance (6/9) of the patients with sequelae, had a moderate disease severity in the acute phase. The large proportion of patients with a moderate disease severity in our study (75%), which is higher than in other TBE studies, (Mickiene et al. 2002; Kaiser 2012), could be at least partly explained by the fact that the study only included hospitalized patients.

The role, and especially the kinetics, of complement activation in TBE needs to be further elucidated. This innate immune response could serve both as a protection towards the viral infection and source of inflammation, which may contribute to the pathogenesis of this infection. In the future, detailed knowledge of the host immune response to TBEV will be essential to facilitate the choice of immunomodulary drugs to be tested together with antivirals.
